# The future of image-guided radiotherapy—is image everything?

**DOI:** 10.1259/bjr.20170894

**Published:** 2018-04-11

**Authors:** David J Noble, Neil G Burnet

**Affiliations:** 1 Department of Oncology, Cancer Research UK VoxTox Research Group, University of Cambridge, Cambridge Biomedical Campus, Addenbrooke’s Hospital, Cambridge, UK; 2 Department of Oncology, Addenbrooke's Hospital, Cambridge University Hospitals NHS Foundation Trust, Cambridge, UK; 3 Division of Cancer Sciences, University of Manchester, Manchester Cancer Research Centre, Manchester Academic Health Science Centre, and The Christie NHS Foundation Trust, Manchester, UK

## Abstract

MR-based image-guided (IG) radiotherapy via all-in-one MR treatment units (MR-linacs) is one of the hottest topics in contemporary radiotherapy research. From ingenious engineering solutions to complex physical problems, researchers have developed machines with the promise of superior image quality, and all the advantages this may confer. Benefits include better tumour visualisation, online adaptation and the potential for image biomarker-based personalised RT. However, it is important to remember that the technical challenges are real. In many instances, they are skillfully managed rather than abolished, a point illustrated by the wide variety of MR-linac designs. The proposed benefits also deserve careful inspection. Better visibility of the primary tumour on an IG scan cannot be bad, but does not automatically equate to better IG, which often depends on a more generalised match to daily anatomy. MR-linac will undoubtedly be a rich milieu to search for IMBs, but these will need to be carefully validated, and similar work with CT-based biomarkers using existing, cheaper, and more widely available hardware is currently ongoing. Online adaptation is an attractive concept, but practicalities are complex, and more work is required to understand which patients will benefit from plan adaptation, and when. Finally, the issue of cost cannot be overlooked, nor can the research community’s responsibilities to global healthcare inequalities. MR-linac is an exciting and ingenious technology, which merits both investment and research. It may not, however, have the future to itself.

A headline debate at this year’s ESTRO congress (2017) was entitled “This house believe that proton guided photons will be superior to photon guided protons”, illustrating that the two “hottest” topics in contemporary radiotherapy research are MRI-guided external beam radiotherapy—MR-linac—and proton beam therapy (PBT). An excellent overview of the theoretical advantages of MR-image guidance, and the capabilities of all-in-one MR treatment units, was recently published in this journal.^1^ This paper eloquently and succinctly describes the physical and engineering challenges of MR-linac. It is clear both from the article and the papers cited that many of these challenges have now been solved, but it is interesting to note that the two major hardware manufacturers initially adopted diametrically opposed principles (cobalt * vs * linear accelerator) in their approach to tackling them. In our view, this serves as a reminder that the physical limitations of MR-linac are real, and should not be ignored. In their introduction, Dr Pollard and colleagues quote Andre Agassi in saying that “Image is everything”, but at least in radiotherapy, this may not be the whole story.

The same introduction^1^ asserts that “the need to ‘see’ the tumour is the basis of image-guided radiotherapy (IGRT)”, a notion we partially challenge. “Seeing the tumour” begins at the time of planning. It is widely accepted that manual target volume segmentation is often the weakest link in the contemporary RT workflow, but this is mainly due to lacking consensus on target volume definition, inadequate training, poor availability of quality imaging for *planning* RT, and intrinsic limitations in diagnostic imaging, including MRI. In other words, inaccurate target volume delineation for planning is not caused by inadequate image guidance (IG) hardware. We suggest that the primary purpose of IGRT is actually to ensure that the planned dose-cube is delivered as accurately as possible to the anatomy of the day, a concept that includes the clinical target volume(s) and organs at risk , as well as the gross tumour volume itself. To our knowledge, there is no evidence as yet that photon-based IG techniques are inferior with regards to this specific endpoint.

The authors rightly suggest that adapting treatment on the basis of imaging biomarkers (IMB’s) is an ultimate objective of MR-guided RT. On this subject, however, we urge caution. As noted in an important recent consensus guideline for IMB studies, approximately 10,000 papers on IMB’s were published between 2004 and 2014, many of these in cancer.^2^ Embarrassingly few have ever been externally validated, and fewer still have changed practice. To paraphrase the conclusions of these papers, IMB’s have great potential to facilitate advances in oncology research and practice, but must first undergo rigorous scrutiny and validation. Furthermore, we challenge the idea that MR is the only IG modality that could be used for this purpose. Whilst MR data sets are obviously a rich environment in which to search for IMB's, it would be fallacious to assume that CT could not be used as well.^3^

Dr Pollard and colleagues initially argue that the raison d’être of MR-IG is superior identification of target structures conferring more accurate image guidance, but subsequently infer that the potential for real-time tracking and modification of RT plans is what really sets MR-linac apart from its competitors. We agree that online plan adaptation is an exciting possibility, but has some reservations about the detail. Firstly, online segmentation of IG images and reoptimisation of dose plans relies on powerful deformable image registration. The accuracy of deformable image registration varies depending on anatomical location, and uncertainties about how best to accumulate dose to organs changing size, shape and position on a daily basis remain. When added to the (admittedly small) uncertainties of dose calculation within a strong magnetic field, we are faced with the possibility that uncertainties in reported differences between planned and accumulated dose may be of a similar magnitude to reported differences themselves.^4, 5^ Even if we accept accumulated dose figures reported by our MR-linac systems entirely at face value, the question of what to do with them remains. Online adaptive RT (ART) is an attractive notion, and will undoubtedly become more widespread in clinical practice over the next decade. However, there is growing consensus that not all patients will benefit from adaptive RT,^6^ or at least that such benefits will be difficult to quantify and measure. Furthermore, we do not yet know which patients will benefit most from plan adaptation, or when is the best time to adapt. MR-IG and MR-linac clearly has a major role to play in answering these questions, but in our view this should be in the research space, rather than widespread clinical use. The co-ordinated and collaborative approach of the Philips–Elekta consortium gives reason for high hope in this regard.^7^

These problems notwithstanding, there is the separate issue of throughput and resource. To our understanding, most current MR-linac workflows require clinician review and “sign-off” of daily segmentation as well as the decision to implement dose reoptimisation, and approval of the new plan. Despite significant (and elegant) automation, this workflow remains highly resource intensive, and would be impractical in achieving a high throughput of patients in many healthcare systems—not least the UK.

This leads onto our next concern, which is, inevitably, one of cost. On the basis of manufacturer figures, we estimate that the cost of replacing sufficient operational linacs in the UK with MR-linac, in order to treat all prostate cancer patients with this technology, to be over £500 million. This is an eye-watering figure in the current economic climate, and the problem is not solely confined to the UK. Parallels with the PBT story in the USA are all too apparent. In 2007, Anthony Zeitmann presciently warned of an impending collision between the Titanic (the shiny new vanguard of high technology—PBT), and the Iceberg (the cold, hard, unyielding laws of economics).^8^ As predicted, American insurance companies in the USA took note of the rapidly rising costs of radiation oncology treatment, and access to PBT for many tumours has been restricted, with predictable consequences.^9^ A key reason for this was the lack of robust clinical data demonstrating clear benefit for the newer, more expensive technology. For MR-linac to succeed, we suggest that coordinated collaboration to generate this evidence must be a priority for the community.^7^ Such research need not only apply to MR-IG and MR-linac; there is much scope for research and development using existing X-ray based systems (especially if manufacturers can improve the quality of current IG-CT), and making the most of, or reaching the “pareto-front” with, the equipment we already have may be a far more cost-effective way of treating the majority of our patients.

Our final point touches on the issue of global inequalities, a problem that is not unique to radiation oncology. It seems that we are following a path of ever-increasing investment into research on increasingly advanced technologies with scope to treat fewer and fewer people. Concurrent with this, there are still countries without a single functioning radiotherapy machine.^10^ Whilst the iniquities of this state of affairs are beyond the scope of this piece, its existence lends credence to the notion that we must do everything possible to optimise our use of the technologies that we already have, and to make them more widely available.^11^

Dr Pollard’s article should be recommended reading for any young radiotherapist wishing to understand the benefits of MR-IG, and the potential it holds for the future. We agree that MR will play an important role in the future of IGRT. It may not be the whole answer, however, and we urge researchers in other areas not to down tools. We cannot side step the issue of cost, and it is imperative that we try to quantify the benefits these technologies confer to our patients. To paraphrase another great tennis player—Arthur Ashe—we should start where we are, use what we have, and do what we can.^12^
